# Agricultural cooperatives participating in vegetable supply chain integration: A case study of a trinity cooperative in China

**DOI:** 10.1371/journal.pone.0253668

**Published:** 2021-06-24

**Authors:** Lu Wang, Jianli Luo, Yuxia Liu

**Affiliations:** 1 School of Business, Wenzhou University, Wenzhou, Zhejiang, P.R.China; 2 Wenzhounese Economy Research Institute, Wenzhou, Zhejiang, P.R.China; Szechenyi Istvan University: Szechenyi Istvan Egyetem, HUNGARY

## Abstract

Supply chain integration plays an important role in the development of the vegetable industry in terms of vegetable quality, vegetable safety, and vegetable security in rural China. This paper explores how agricultural cooperatives integrate the vegetable supply chain by taking a trinity cooperative as an example in China. It explains the translation concatenation of supply chain integration for this cooperative by constructing actor networks in four development stages, including the seed stage, start-up stage, development stage, and mature stage. The findings show that supply chain integration in production cooperation, supply & sales cooperation, and credit cooperation is a useful trinity cooperative model of supply chain integration for investigating vegetable supply chain integration through internal integration and external integration. This paper suggests that cooperatives in the vegetable supply chain should facilitate close coordination among different shareholders and further improve the efficiency of supply chain integration. The government should provide training opportunities and funding to encourage cooperatives to participate in supply chain integration within the vegetable industry.

## 1 Introduction

Rapid growth in vegetable consumption and scarce land resources with a large population has increased the pressure to redesign the current supply chain to encompass economic, social, and environmental views in rural China [1]. An increasing number of people are concerned about vegetable quality, vegetable safety, and vegetable security [2], resulting in an urgent need for farmers to integrate the vegetable supply chain. In addition, in 2018, the Chinese State Council enacted a policy meant to shift the agricultural supply chain from a traditional agricultural circulation to a modern one through updates in standardization, intelligence, coordination, and greenization. This represents an opportunity to reform the supply chain in the agricultural industry. To improve vegetable quality, vegetable safety, and vegetable security, it is crucial to emphasize supply chain integration by not only facilitating cooperation among different sectors in the vegetable supply chain but also improving the operational performance of vegetable production, supply, and sales within it. There are many types of vegetable supply chains in rural China. Based on the dominant position, the current supply chain model in the vegetable industry consists of a large-scale chain supermarket-dominated, a wholesale market-dominated, and production & processing enterprises-dominated supply chain in China.

There are some challenges preventing vegetable supply chain sustainability in rural China. One challenge is neglecting farmers’ benefits [3]. Similar to farmers in South Korea and Denmark [4], most Chinese vegetable producers are small-scale or family-oriented farmers. The three types of vegetable supply chains mentioned above (large-scale chain supermarket-dominated, wholesale market-dominated, and production & processing enterprises-dominated) ignore small-scale farmers’ benefits by transferring them to large companies such as large-scale chain supermarkets, wholesale markets, and production & processing enterprises. Other challenges exist within the pre-, during, and post vegetable production processes, comprising a technology shortage in vegetable production, storage, and preservation [5]; uncertainty in vegetable supply and sales [1, 6]; and imperfect rural financial support [7]. It is not easy for large-scale chain supermarkets, wholesale markets, and production & processing enterprises to control the pre-, during, and post vegetable production processes. Thus, vegetable supply chains such as the large-scale supermarket-dominated, wholesale market-dominated, and production & processing enterprise-dominated have difficulties solving these challenges.

Similar to Asian countries such as South Korea and European countries like Denmark, agricultural cooperatives play significant roles in the agricultural economy [4, 8] and impact rural development in China [5, 9]. To address these challenges, building agricultural cooperatives allows small-scale farmers to increase their vegetable price bargaining power in the vegetable market [6]. Moreover, it is also easy for agricultural cooperatives to govern farmers in pre-, during, and post production processes. For example, agricultural cooperatives can collectively purchase agricultural materials such as seeds, which contribute to a transaction cost reduction. Agricultural cooperatives participating in the vegetable supply chain are more important during the current anti-coronavirus era because this supply chain greatly reduces the risk of infection between producers and consumers. To stop the spread of the virus, China encouraged no-touch delivery, which had a significant impact on the vegetable supply chain. The vegetable industry throughout China experienced disruptions to vegetable supply chains due to COVID-19, whereby the countermeasure of cooperatives participating in the supply chain attempted to resolve these disruptions. It was relatively easy for agricultural cooperatives to establish vegetable supply chain integration and integrate no-touch delivery through small-scale farmer coordination and information sharing. Thus, it is important for agricultural cooperatives to participate in the vegetable supply chain to minimize the negative economic impacts of COVID-19 on the vegetable industry and the risks that may arise as a result. The research questions can therefore be listed as:

1. How do agricultural cooperatives participating in the vegetable supply chain integrate supply chains in the vegetable sector?
2. What actions do agricultural cooperative operations perform based on supply chain integration?

To resolve the above two research questions, this research uses the case of the Meiyu Cooperative in the vegetable supply chain in China to examine how agricultural cooperatives participating in supply chain integration, in order to facilitate supply chain partnerships within the vegetable sector, collaborate to conduct a trinity cooperative mode of supply chain integration. Theories regarding agricultural cooperatives in China, actor network theory, and relevant supply chain theories are explained in the literature review section. Then, this study illustrates the case study method and the data collection method used in the case of the Meiyu Cooperative in section 3. Next, we reveal the Meiyu Cooperative’s supply chain integration—how it shifted from conflict to coordination among shareholders through the four developmental steps of seed stage, start-up stage, development stage, and mature stage—by using actor network theory. Finally, this study will highlight how the relationship between supply chain coordination in production, supply & sale, and credit in the Meiyu Cooperative establishes a trinity mechanism that is useful for internal and external integration in the vegetable sector in China.

## 2 Literature review

### 2.1 Agricultural cooperatives in China

Agricultural cooperatives have contributed greatly to economic growth as well as social and environmental development in rural China, especially after the implementation of the Farmers’ Professional Cooperative Law in 2006 [5]. According to statistical data from the Minister of Agriculture and Rural Affairs (MOARA), the number of registered farmer agricultural cooperatives reached 2.21 million in 2019. MOARA also claimed that approximately 50% of farmers in China were covered by farmer agricultural cooperatives as of 2019. Of those, more than half are part of the fruit and vegetable industry.

Agricultural cooperatives continue to enrich organizational forms, continuously expand the fields of industry, and constantly expand the industrial supply chain, affirming their leading role in agricultural development [4, 10]. As a study shows that the agricultural cooperative is a vehicle to reach diverse purposes [8]. For instance, agricultural cooperatives expand their service functions in vegetable seed supply, collective purchases of agricultural materials, production management, production processing, and sales, all of which help small-scale farmers increase revenues [11]. The multilevel governance of agricultural cooperatives in Europe in particular promotes competition in the global agricultural market [8].

The key for agricultural cooperatives in China is each member’s equal voting rights, supported by the Farmers’ Professional Cooperative Law [5], which encourages farmers to attend agricultural cooperatives. As an information mediator, agricultural cooperatives provide valuable information to their members [12]. Thus, the information sharing through joint problem-solving in agricultural cooperatives promotes and establishes social trust among farmers [13]. Partnerships based on faith in loyalty and capability are high among farmers in agricultural cooperatives, facilitating efficient and sustainable agriculture [12, 14], while moral hazards destroy trust, eroding farmers’ willingness to cooperate [15]. Trust among members is therefore an important predictor of group cohesion and further strengthens members’ desire to remain in agricultural cooperatives [16]. More importantly, farmers’ participation and cooperation in agricultural cooperatives help them improve the adoption of production technology [17–19], increase their revenues [14], and overcome social barriers [20]. Thus, agricultural cooperatives contribute to rural sustainable development in China.

### 2.2 Actor network theory

Actor network theory describes how various actors establish alliances and involve other human and nonhuman actors to secure their interests [21]. For example, Studies focuses on different actors’ exchange actions within the actor network [22], which determines the community of interest coordination from different backgrounds. Researchers believe that the products, values, and actions of various actors are different because the actor network is organized by different actors [23]. The actor network is based on different interests from different actors [23]. Chinese scholars focus on solving real problems with actor network theory, such as communication standardized strategies [24], the mechanism of knowledge production for interdisciplinary innovation teams [25], and strategies for regional innovation [26].

The key to using actor network theory is to mobilize various resources from different actors, including humans and nonhumans, into the action governing through the mechanism of translation concatenation, securing their interests to resolve problems. The mechanism of translation concatenation usually moves through four stages of translation processes: problematization (focal actor defines other actors’ interests and suggests how these interests would be resolved), interessement (focal actor makes itself indispensable to other actors and defines other actors’ interests), enrollment (other actors accept the defined interests and are involved in the actor network), and mobilization (the focal actor ensures other actors’ behaviors and interests based on their agreement) [21, 27, 28]. The actor network is organized by its different actors, whereby the key for network establishment is the integration of various interests by different actors through their concentrating discrete objectives into a common aim via coordination. Problems can be resolved by obligatory passing points that are negotiated by different actors [28] (common interest mobilized by different actors). The focal actor plays an important role in this actor network by defining and ensuring other actors’ behavior and interests.

### 2.3 Supply chain integration

Supply chain integration refers to strategic alliances across different sectors in supply chains [29]. The purpose of supply chain integration is to facilitate inter- and intraorganizational governance processes [3, 30]. Vegetable supply chain integration has been commonly used in research and includes shareholders’ relationships, contracts, and strategic alliances, as well as internal and external integration [2, 3, 31]. This means that various shareholders—e.g., vegetable material suppliers, vegetable producers (farmers), institutional vegetable retailers, banks, and vegetable customers—collaborate and integrate in the vegetable industry in China. Supply chain performance can be improved by emphasizing the importance of quality, technology, and logistics for agricultural cooperatives [17]. To resolve problems such as costs, risk, and performance, supply chain integration requires coordination with involved shareholders and can be classified into two types: internal integration and external integration [30, 32–34]. Coordination among shareholders in the supply chain of the agricultural industry facilitates economic, social, and environmental standards through effective communication between farmers and other sectors [35].

Internal integration can be defined as the degree to which enterprises transfer internal strategies, actions, and procedures to a cooperative and consistent process in order to rapidly meet customers’ demands and communicate with other partners [30, 32, 34]. Internal integration consists of information sharing, data and process coordination, and cooperation among internal departments [30, 34]. External integration is the extent to which organizations administer intrastrategies, actions, and procedures at a collaborative and manageable level to conduct strategic alliances with suppliers, customers, and other partners [34]. External integration includes coordination with customers, suppliers, and other partners. It consists of information sharing, strategic alliance, consistent planning, and collaboration with customers, suppliers, and other partners [32]. The coordination of internal and external integration contributes to the supply chain management process [2]. Internal integration positively impacts external integration within the operation implementation process [36]. According to the literature, there are several outcomes produced by supply chain integration, such as strengthening the relationships between different sectors in supply chains [31], and improving economic, [7, 17], social [5], and environmental benefits [37]. The Meiyu Cooperative is a trinity cooperative. We suggest that the Meiyu Cooperative in China has a high level of internal integration that not only acquires similar benefits from coordination but also integrates its external networks and shares its benefits with supply chain shareholders to realize operational performance improvement.

This paper studies the Meiyu Cooperative’s participation in supply chain integration by using a translation mechanism from actor network theory. Specifically, this study investigates the modes of different actors’ cooperation and combination through their actions. More importantly, this paper analyzes the Meiyu Cooperative’s supply chain integration from a dynamic perspective, including the different stages of its internal and external integration.

## 3 Methodology

### 3.1 Case study method

The case study is an in-depth investigation that dissects, analyzes, and summarizes typical phenomena to inspire new theories and new methods [38]. It can help expand the application scope of special conclusions in different situations and provide practical suggestions for related topics. The case study method is suitable for this research, which explores a trinity mechanism of supply chain integration by investigating the Meiyu Cooperative through actor network theory. This theory is an original method that is appropriate for a qualitative assessment of the Meiyu Cooperative’s networking process. Our major research interest rests in how actor networks are conducted; that is, using actor network theory to comprehend how various actors in the supply chain develop integration through their involvement in complex networks of different associations.

### 3.2 Data collection

To guarantee the variety of data resources, first-hand data from interviews and second-hand data from the Meiyu Cooperative’s official website were collected. The Ethics Committee, School of Business, Wenzhou University specifically approved this study. Following one researcher’s ideas [38], to completely understand the development process of the cooperative, we conducted six face-to-face in-depth interviews with three chairmen, one clerk, and two members through designed research questions and outlines. Each interview lasted for two hours. Then, three researchers sorted out the interview transcripts within 24 hours and double-checked issues such as a vague expression or unclear information through follow-up interviews by phone. All interviews were completed from August 2017 to January 2018. The individuals interviews in Meiyu Cooperatives have provided written informed consent (as outlined in PLOS consent form) to publish their interviews alongside the manuscript.

### 3.3 Description of the Meiyu Cooperative

Located in Wenzhou City, Zhejiang Province, the Meiyu Cooperative is one of the top 100 national farmers’ cooperatives in China. This cooperative consists of 762 members involving 41 villages and seven towns in Wenzhou City. According to the Provincial Statistic Bureau’s data, its members’ annual revenue was USD 11,160 in 2019 (almost 10% higher than the average annual per capita GDP of $10,145 USD of the local country). As one of largest provincial vegetable production bases, Meiyu has approximately 1,300 acres of land, contributing nearly USD 15,600,000 of annual economic output. Approximately 60% of vegetables in this cooperative are supplied to local cities, with 40% sold elsewhere. As a leading agricultural cooperative in China, the Meiyu Cooperative feels a heightened sense of social responsibility concerning environmental protection and vegetable security.

The Meiyu Cooperative’s development can be divided into four stages: seed stage, start-up stage, development stage, and mature stage. In the seed stage from 1991 to 2003, some farmers cooperated in production, received support from agricultural institutions to guarantee fresh vegetables, and expanded business in vegetable production. This cooperative founded several famous fresh vegetable brands, such as “Qianglv” (striving green) and “Lvyinxiang” (green impression), and developed into one of the top cooperatives of Zhejiang Province. In the start-up stage (2004–2008), the Meiyu Cooperative focused on supply cooperation in the supply chain through the Supply Agency. For instance, the Supply Agency introduced new varieties of vegetable seeds from Israel. The Meiyu Cooperative paid special attention to cooperation in vegetable sales during the development stage (2009–2011) through the Wanke Company. For example, it cooperated with wholesale vegetable markets by agreements to build 13 sale points around China. Finally, it emphasized credit coordination within the cooperative to increase financial integration in the mature stage (2011–2020) through the Credit Agency called the Huimin Rural Mutual Funds Union. By integrating production cooperation, supply and sales cooperation, and financial credit cooperation during these four development stages, the Meiyu Cooperative fostered collaboration among different shareholders to facilitate the effectiveness of the vegetable supply chain and improve the development of agricultural cooperatives. Moreover, the cooperative achieved economic, social, and environmental benefits through farmers’ revenue increase, community social welfare improvements, and better local environmental protections.

### 3.4 Data analysis

Three researchers analyzed the interview transcripts, archival data such as annual reports, other data from official websites, and direct observations. Implementing one study’s triangulation method for different resources [39], this study created and profiled three databases, including the Development Stages of Meiyu Cooperative Database, The Actor Network Constitution of Meiyu Cooperative Database, and the Trinity Meiyu Cooperative in Supply Chain Integration Mode Database. Three researchers sorted out the case data and then coded the information from quantitative data to non-quantitative data. Then, we returned to check with our interviewers and clarified the coded data if the conclusions conflicted. Through clearing up, profiling, and mutual testing, the results of the case study from different resources showed consistency, which guaranteed reliability among coders. Disagreements were also double-checked in this way. Based on the data analysis, researchers achieved a broad understanding of the trinity Meiyu Cooperative supply chain integration mode and how the actor network functions within this cooperative.

## 4 Findings

### 4.1 Actor network structure for the Meiyu Cooperative

The actor network formation of the Meiyu Cooperative requires different actors in the four development stages to coordinate and integrate. As a focal actor in the actor network, the Meiyu Cooperative worked together with other actors to solve its and others’ problems with obligation passage points: in the seed stage, a lack of production technology required the Meiyu Cooperative to cooperate with institutions to improve its vegetable production skills; the high cost of purchasing production materials compelled the Meiyu Cooperative to coordinate with vegetable supply companies to establish the Supply Agency during the start-up stage; the shortage of sale channels demanded that the Meiyu Cooperative work closely with wholesale markets and supermarkets to expand its market in the development stage; and the shortage of financial resources forced the Meiyu Cooperative to coordinate with banks during the mature stage. To establish the obligatory passing points and overcome its problems, the Meiyu Cooperative integrated different actors and prioritized other actors’ interests (such as helping farmers increase revenues, raising operation revenues for retailers, aggrandizing the number of customers, and facilitating vegetable safety for consumers). Therefore, actor network formation needs clear actors and problems during different network development stages. The focal actor integrates different actors to establish obligatory passing points through translation concatenation within the actor network.

**Fig 1:**
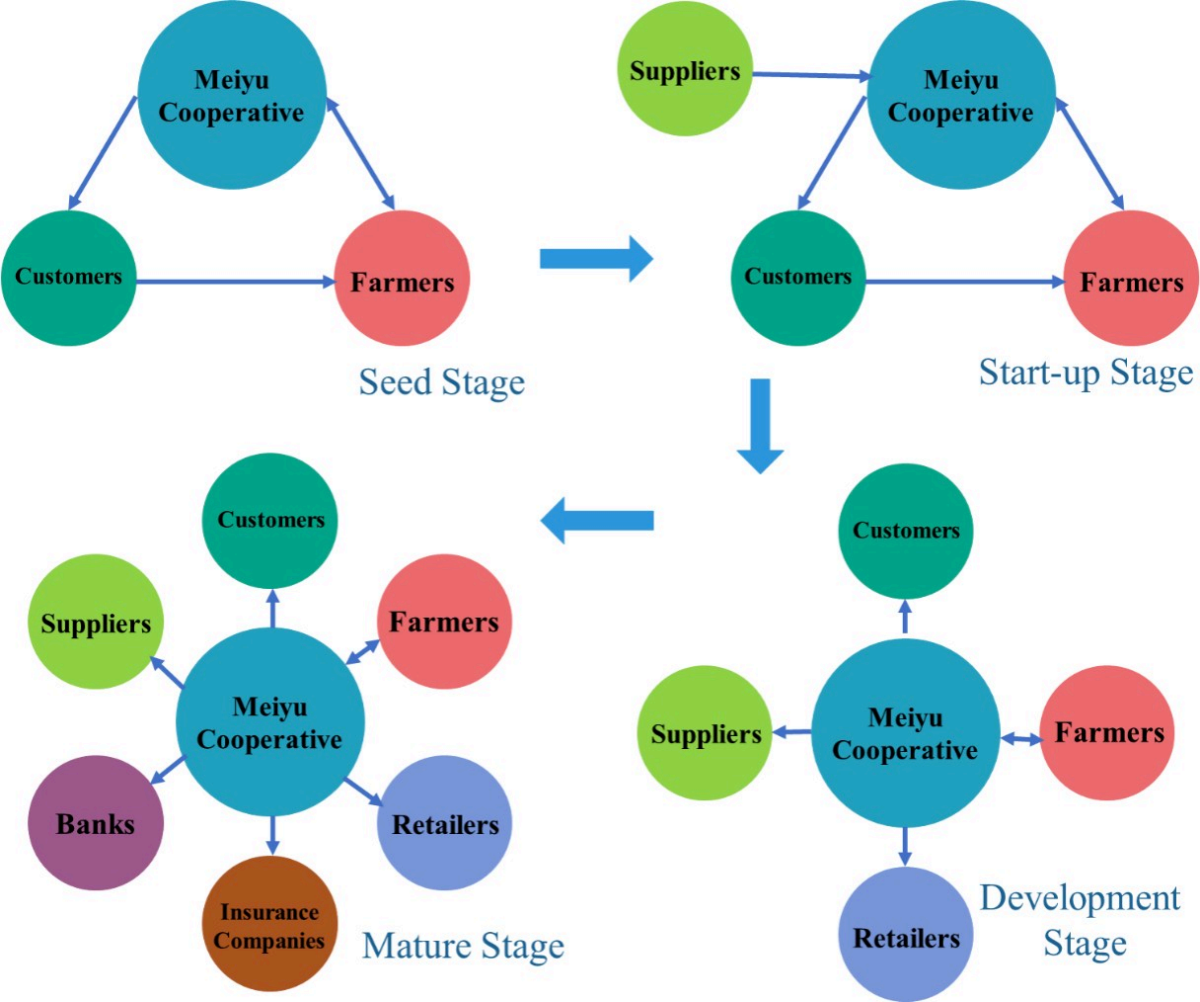
The changes in the network actors in the Meiyu Cooperative.

First, various actors in the Meiyu Cooperative supply chain are clarified. The actors in the Meiyu Cooperative supply chain change during the four stages (Fig 1), including the Meiyu Cooperative, farmers, consumers, suppliers (e.g., supply companies), retailers (e.g., wholesale markets and supermarkets), banks, and insurance companies.

**Fig 2:**
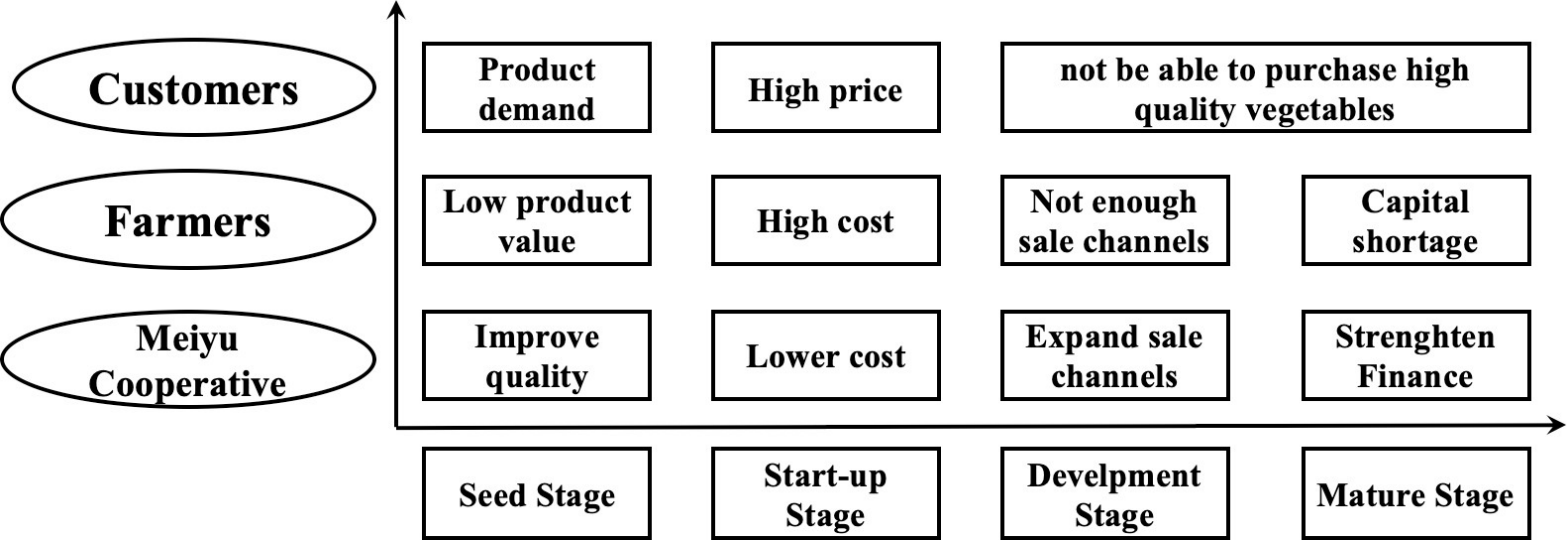
The Meiyu Cooperative’s, farmers’, and customers’ problematization in different stages.

Second, problems in different stages are defined. Problems in the actor network change along with the four stages in the Meiyu Cooperative. Problems emerge for consumers, farmers, and the Meiyu Cooperative (Fig 2). The problems’ evolutionary process in the Meiyu Cooperative are as follows: the Meiyu Cooperative develops along with customers’ demands for vegetables; due to the problems of high-quality vegetable demand from customers and the vegetable production improvement demand from farmers, the Meiyu Cooperative must establish the Production Agency to facilitate production skill and meet customer and farmer demand in the seed stage; the problems in the start-up stage are the high vegetable price for customers and high cost of purchasing agricultural materials for farmers, whereby the Meiyu Cooperative has to consider strategies of lower cost in supply; in the development stage, customers cannot buy the high-quality vegetables and farmers have a shortage of sale channels, so it is important for the Meiyu Cooperative to expand sale channels and stabilize sales; in the mature stage, the problems of dissatisfactory customer experience and shortage of capital require the Meiyu Cooperative to pay more attention to attracting inflows of capital.

**Fig 3:**
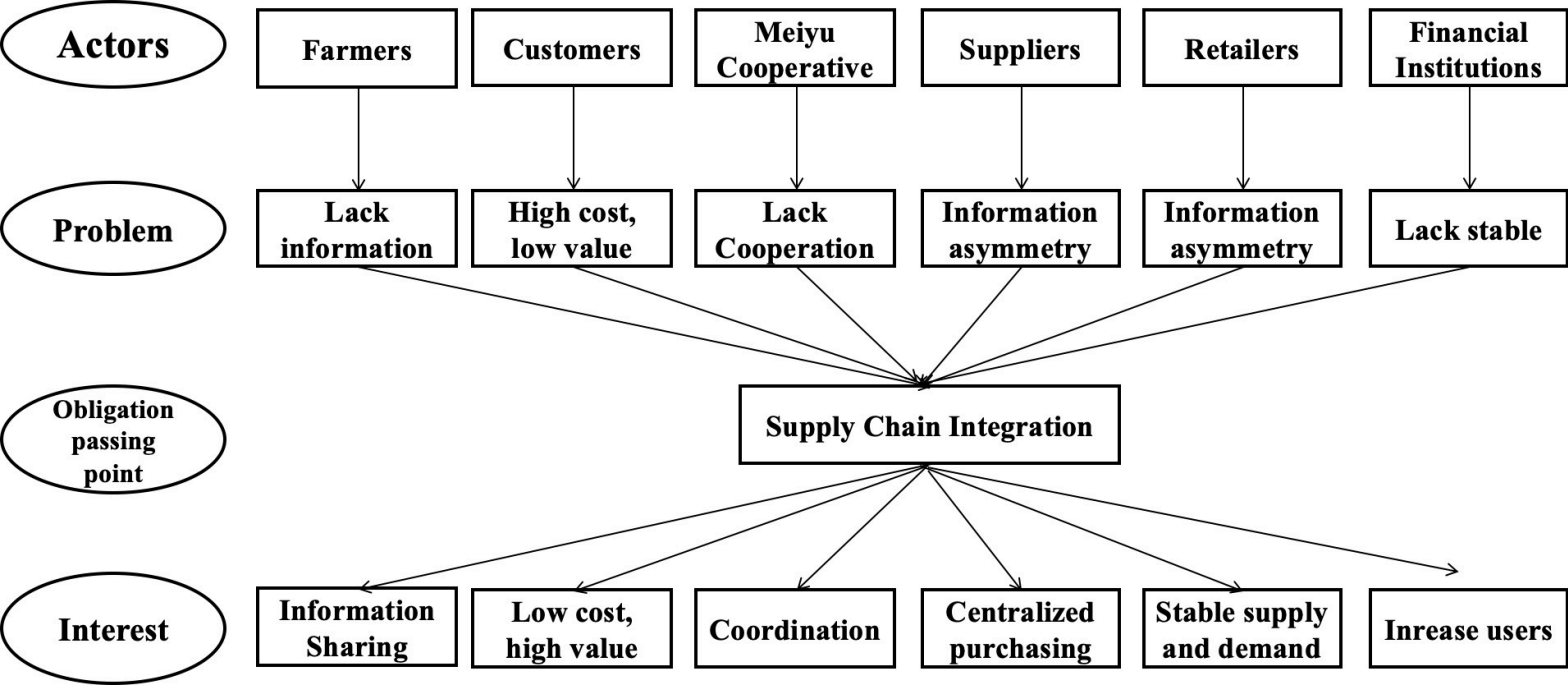
Supply chain integration obligatory passing point for the Meiyu Cooperative.

Finally, obligatory passing points are negotiated to solve problems in an actor network. The Meiyu Cooperative is the focal actor in the actor network. Based on the problems defined by other actors in the network, supply chain integration can function as the obligatory passing point to solve problems. By forming the actor network to include farmers, customers, suppliers, retailers, banks, and distributors, the Meiyu Cooperative uses supply chain integration as the obligation passage point for various actors to solve problems and gain interests that are then accepted by other actors. This cooperative positively attracts different actors to participate in the actor network and engage in alliance collaboration among themselves, which in turn compels them to depend on the cooperative. The Meiyu Cooperative collects all problems to be resolved in obligatory passing points and then mobilizes various resources through supply chain integration to share interests with different actors (see Fig 3). Other actors are actively involved in the actor network to coordinate bettering their interests according to agreements that they will not betray.

### 4.2 Supply chain integration of the Meiyu Cooperative

**Fig 4:**
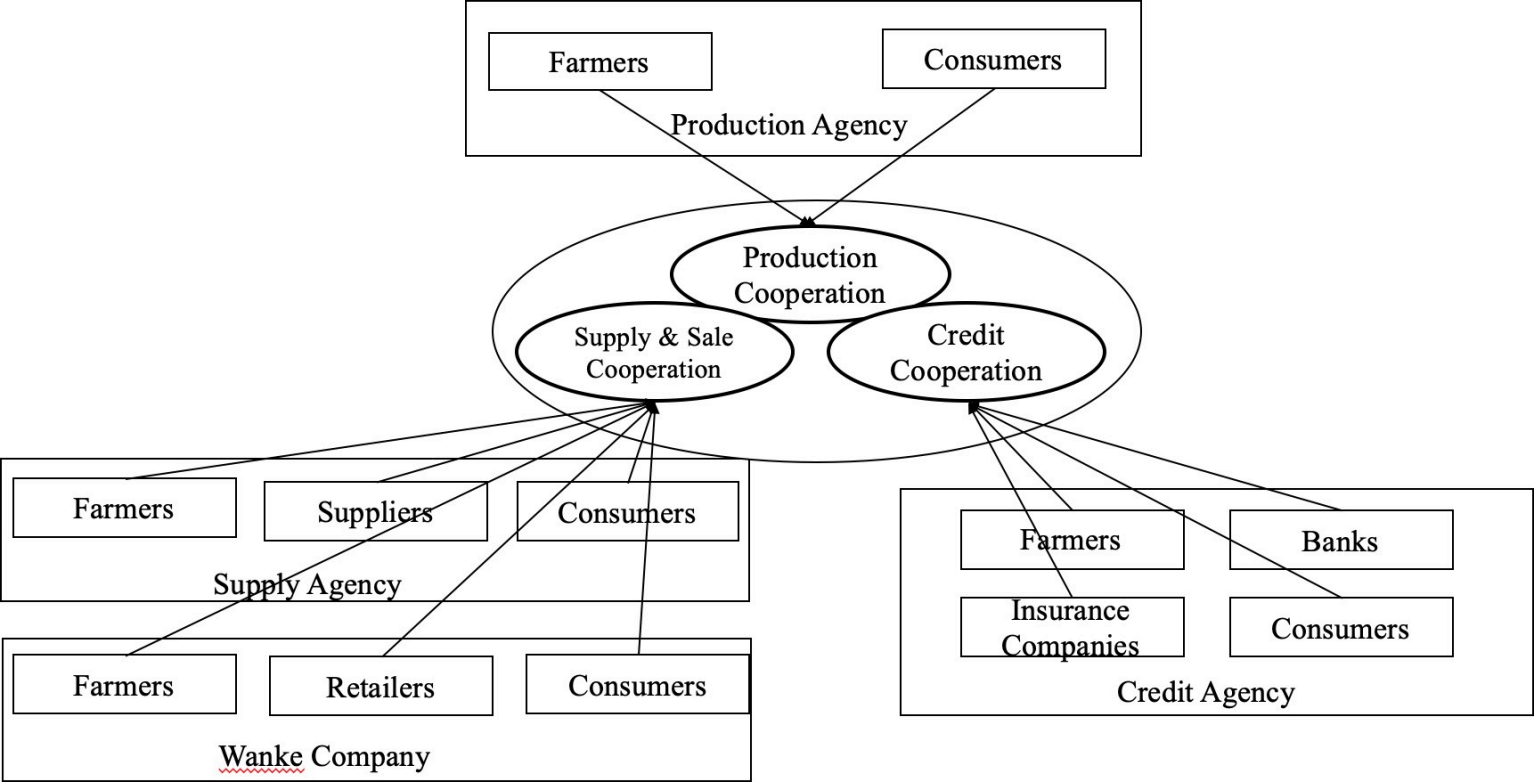
The Meiyu Cooperative’s trinity supply chain mode.

According to the actor network theory, shareholders who participate in the development process of the Meiyu Cooperative’s supply chain integration are the actors—the Meiyu Cooperative, farmers, suppliers, retailers, banks, and customers. These shareholders establish strategic alliances by interests and allocate the resources to go through translation concatenation, which collects dispersive resources concentrated to resolve problems during the four different stages of the Meiyu Cooperative and further facilitates ensuring the aims of supply chain integration. Therefore, different sectors in the Meiyu Cooperative could coordinate with each other to establish an actor network, improve efficiency, and further realize the aims of supply chain integration. This study investigates actors’ motivations, actions, and effects on supply chain integration by tracking the behaviors of the Meiyu Cooperative at all development stages and deciphering the complex interaction and combination patterns of the actors in the actor network. More importantly, the trinity supply chain mechanism for the Meiyu Cooperative transforms traditional supply chain management to modern supply chain integration. This means that the Meiyu Cooperative integrates supply chains with production cooperation, supply & sales cooperation, and credit cooperation through a strategic alliance with shareholders in supply chains (Fig 4).

#### 4.2.1 Seed stage: Production cooperation

**Fig 5:**
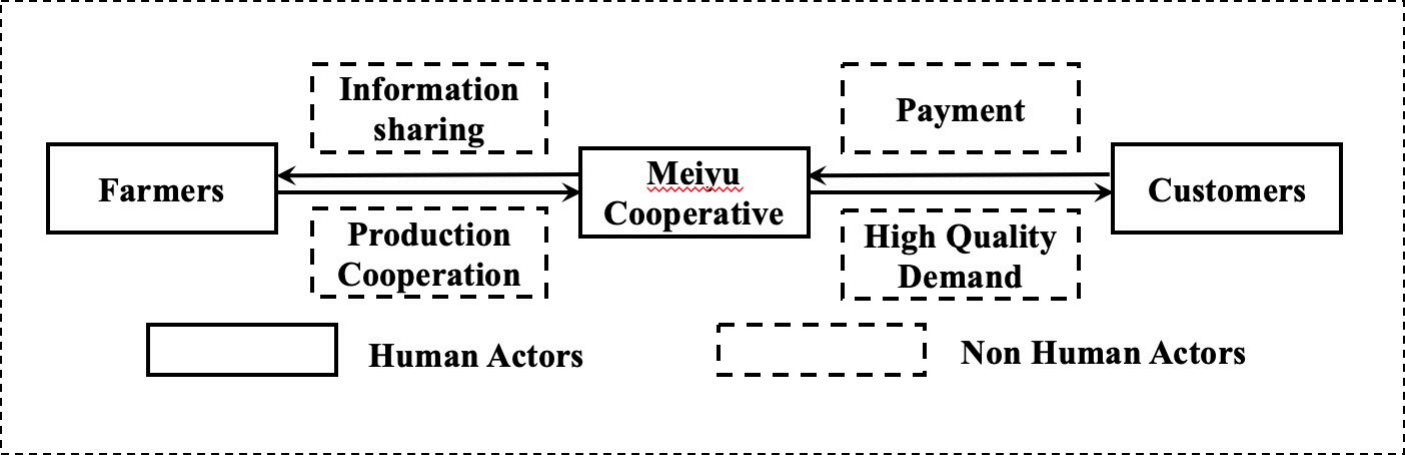
Seed stage.

In the seed stage, to meet customers’ high-quality vegetable demand and farmers’ high-skill vegetable demand, the Meiyu Cooperative founded Production Agencies such as the Vegetable Technology Service Center and Agricultural Residue Testing Center to provide production skills support for farmers via production cooperation (Fig 5). In this stage, the Meiyu Cooperative defines other actors, such as customers and farmers, in the actor network and confirms “interessement” through agencies to improve production cooperation. Therefore, the Meiyu Cooperative has successfully completed the “problematization” validity and cooperated with the other actors suggested by this cooperative.

Hence, the Vegetable Technology Service Center helped farmers lower the production cost through a technology provision. Additionally, the efficient use of fertilizer, pesticide, and the quality control of vegetables were assured by the Agricultural Residue Testing Center. The Meiyu Cooperative therefore successfully realized the translation process of “interessement” in the translation concatenation of supply chain integration to match the “problematization”.

This cooperative established the Production Agency to perform supply chain integration in production and establish an obligatory passing point for other actors’ achievement of gains. The Meiyu Cooperative effectively fulfilled the interests of various actors that the cooperative had previously defined for them. The Meiyu Cooperative could thus attract other actors—such as institutions participating in the supply chain integration actor network during the “enrollment” process—through the Production Agency. For instance, with the assistance of agricultural institutions, the cooperative applied galvanized steel pipe skills to establish greenhouses, ensuring vegetable production over cold winters.

Finally, the Meiyu Cooperative mobilized different human and nonhuman actors to initially construct a stable actor network and complete the “mobilization”. The Meiyu Cooperative has produced many famous fresh vegetable brands to guarantee high-quality vegetables, which in turn helped it become more competitive in the retail market. In other words, the Meiyu Cooperative improved vegetable quality and lowered cost through the involvement alliance of different actors, such as farmers, customers, and institutions, in the actor network based on production integration during the seed stage.

Through actor network formation in the seed stage, different actors in the Meiyu Cooperative’s actor network increase coordination with each other. This close relationship plays a vital role in improving internal integration during the vegetable production process. The Meiyu Cooperative conducts the internal integration of information sharing between farmers in production through the Production Agency to facilitate production skills. Additionally, by collaborating across different functions, such as agricultural institutions in the production process, the Meiyu Cooperative facilitates external integration and improving production skills. The Meiyu Cooperative’s internal integration and external integration in production recognize that different functions within a cooperative should belong to the process of integration. Through internal integration and external integration in production, the Meiyu Cooperative has effectively met customers’ demand for high-quality vegetables.

#### 4.2.2 Start-up stage and development stage: Supply and sales cooperation

**Fig 6:**
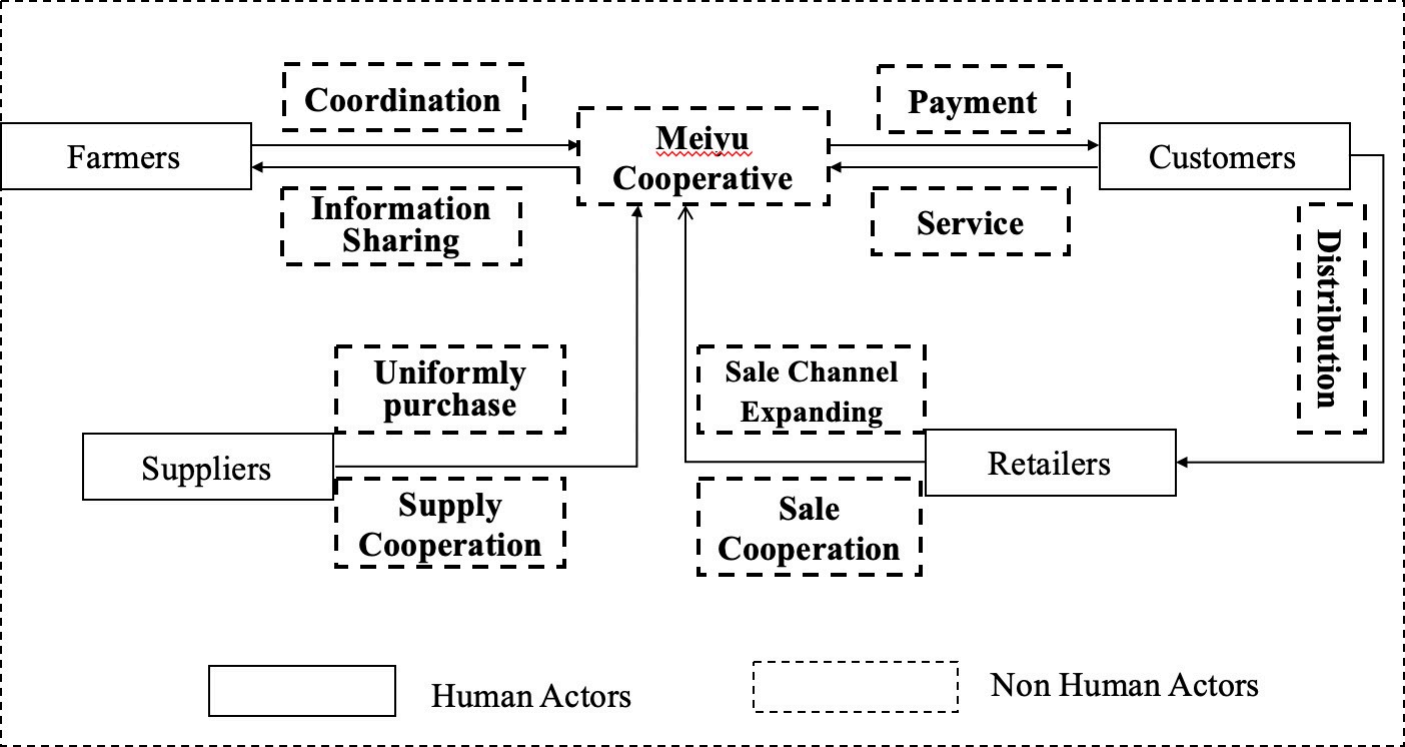
Start-up stage and development stage.

In the start-up and development stages (see Fig 6), the focal actor of the Meiyu Cooperative and other actors, such as customers, suppliers, retailers, and farmers who want to participate in supply and sales cooperation, must confirm “problematization”. In addition, the quality of vegetables is guaranteed. However, farmers cannot purchase agricultural resources at a low cost and have difficulties expanding the sales market and selling their vegetables at a reasonable price; customers cannot buy high-quality vegetables at a reasonable price. Therefore, “problematization” is defined by the Meiyu Cooperative and other actors.

After “problematization”, the Meiyu Cooperative supports farmers in supply and sales cooperation to confirm “interessement”. Specifically, the Supply Agency helps farmers lower the agricultural material purchasing cost through a unified sourcing and a large scale of supply. In addition, market channels (e.g., wholesale markets, supermarkets, and schools) are expanded, and the negotiation of market prices with markets is guaranteed by the Wanke Company. Thus, the translation process of “interessement” successfully ensures the validity of the “problematization”.

The Meiyu Cooperative established the Supply Agency and the Wanke Company to perform supply and sales integration and establish an obligatory passing point for achieving other actors’ interests. The Meiyu Cooperative is then able to fulfill the interests of various actors, and others also accept the interests that this cooperative previously defined. The Meiyu Cooperative can attract various actors to participate in the supply chain integration actor network in the “enrollment” process through the Supply Agency and the Wanke Company.

To establish a firm actor network and complete the “mobilization” in the final process of translation, the Meiyu Cooperative involves different human and nonhuman actors. For instance, with the assistance of agricultural material companies, this cooperative collectively purchased seeds and fertilizer at a reasonable price with supply cooperation. Another example is that many companies, such as Baihui Supermarket, signed a sales contract with the Meiyu Cooperative to purchase fresh vegetables in advance. In other words, the Meiyu Cooperative lowers the vegetable cost and expands sale channels with the involvement of an alliance of various human actors (e.g., farmers, customers, agricultural material companies, supermarkets, schools, and wholesale markets) and nonhuman actors (e.g., supply services and sales contracts) in the actor network based on supply and sales integration in the start-up and development stages.

During the start-up stage, the Meiyu Cooperative conducted internal integration with farmers in supply through the Supply Agency, collectively purchasing agricultural materials such as fertilizer at a comparatively low price. Additionally, with a less unified use of antibiotics from suppliers, the Meiyu Cooperative ensured vegetable quality. To achieve a high degree of integration with suppliers and customers in the supply chain, the Meiyu Cooperative formed external integration with stabilized agricultural material supplier companies through strategic alliances of supply agreements in supply cooperation. Employing internal integration and external integration in the supply sector, the Meiyu Cooperative not only lowers vegetable costs to increase farmers’ revenues but also lowers vegetable prices, which benefits vegetable consumers.

The Meiyu Cooperative accomplished internal integration with farmers in sales through the Wanke Company and unified sales during the development stage. Moreover, the Meiyu Cooperative also established external integration with other partners through sale agreements. For example, a restaurant in Wenzhou Middle School placed orders with this cooperative to buy fresh vegetables for students and teachers in advance through a sale contract. Through internal integration in sales, the Meiyu Cooperative achieved a competitive advantage of vegetables in the vegetable market. More importantly, the Meiyu Cooperative formed a collaborative relationship with retailers and increased farmers’ price bargaining power through external integration in sales. In addition, retailers stabilized fresh vegetables for customers.

#### 4.2.3 Mature stage: Credit cooperation

Issues concerning the quality of vegetables, low-cost agricultural material purchasing, and a reasonable market price are guaranteed in the development stage (Fig 6). However, farmers have difficulties obtaining financial support from local banks due to their limited credit; customers cannot buy high-quality vegetables promptly at a reasonable price. The Meiyu Cooperative became necessary to negotiate with other actors regarding the problems defined above and then suggested that these issues would be resolved once actors negotiated the obligatory passing point of credit cooperation.

Here, the Meiyu Cooperative ensured “interessement” through the Credit Agency to improve financial credit cooperation among different actors. It established the Huimin Rural Mutual Fund Union and the Rui’an Xingmin Rural Insurance Mutual Aid Association to perform financial integration and establish an obligatory passing point for achieving other actors’ interests. Thus, the Meiyu Cooperative successfully achieves the translation process of “interessement” in the translation concatenation of supply chain integration to match “problematization”. Moreover, the validity of the “problematization” is clarified.

Next, the Meiyu Cooperative addressed the interests of various actors that this cooperative defined in advance. The Meiyu Cooperative can attract various actors to become involved in the financial credit integration actor network in the “enrollment” process through the Huimin Rural Mutual Fund Union. The Credit Agency helps farmers increase the efficiency of capital and gain financial support.

**Fig 7:**
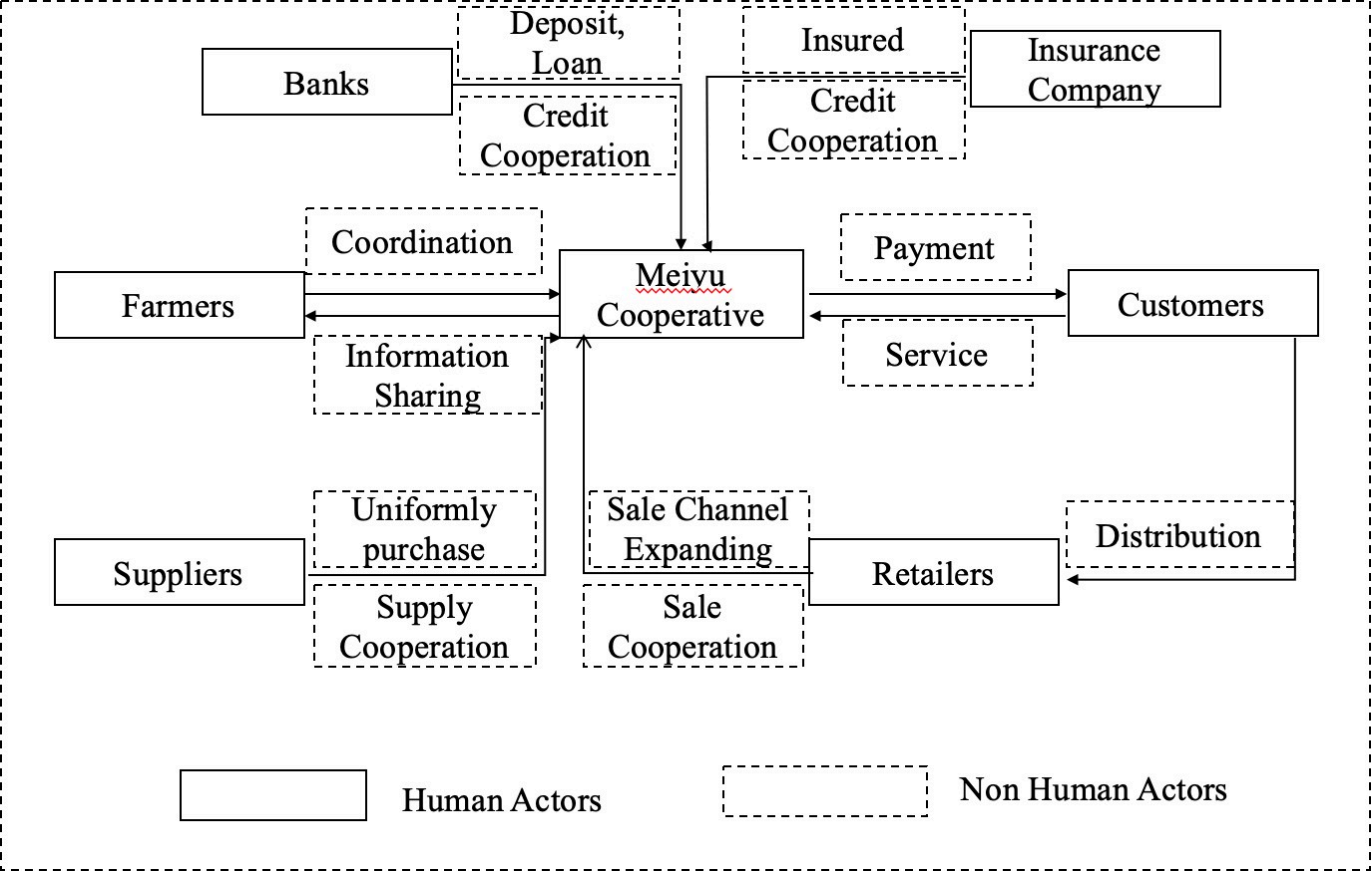
Mature stage.

Through the final translation process of “mobilization”, the Meiyu Cooperative mobilizes different human and nonhuman actors to initially establish a solid actor network according to their agreements (Fig 7). For instance, this cooperative can solve the problem of the lack of capital to purchase agricultural materials based on credit agreements through cooperation with banks. Another example is that farmers insure greenhouse tomato overwinter to prevent natural disasters for under USD 185 per Chinese acre (approximately 0.16 acres) through contracts within insurance companies and insurance support from the Rui’an Xingmin Rural Insurance Mutual Aid Association. In other words, the Meiyu Cooperative increases the capital capability using the involvement alliance of various human actors (e.g., farmers, customers, banks, and insurance companies) and nonhuman actors (e.g., financial support and insurance contracts) in the actor network based on financial credit integration during the mature stage.

In the mature stage, the Meiyu Cooperative established internal financial credit integration with farmers through the Huimin Rural Mutual Fund Union (based on a high level of internal communication ability) and external financial credit integration with banks and insurance companies (based on a high degree of external coordination ability). For internal financial credit integration, with the close coordination of partners in advance, farmers voluntarily attend and are free to exit the distribution of profits from the Huimin Rural Mutual Union. This union assists farmers with financial services such as deposits, loans, and settlement in the Meiyu Cooperative, which facilitates the efficiency of capital flows between farmers and the cooperative. To achieve a high level of external credit integration, the Meiyu Cooperative cooperated with Rui’an Rural Commercial Bank through a credit contract of USD 1,153,800. According to the contract, farmers in this cooperative can borrow money directly from the bank based on their agricultural needs in the pre-, during, and postproduction processes. For instance, farmers can purchase greenhouses to produce tomatoes during winter in advance with loans from Rui’an Rural Commercial Bank, and the Meiyu Cooperative would then return the funds to the bank through selling tomatoes based on agreements with farmers. This external financial credit integration improves financial flows among farmers, the Meiyu Cooperative, banks, and retailers. Another example of external financial credit integration is participation in the Rui’an Xingmin Rural Insurance Mutual Aid Association. From this external insurance cooperation, farmers experience a timely settlement of claims, allowing them to more swiftly return to vegetable production following a loss. Furthermore, farmers can increase their credit value by purchasing insurance and obtaining more securities. This external financial credit integration reduces risks among farmers, cooperatives, and insurance companies.

**Fig 8:**
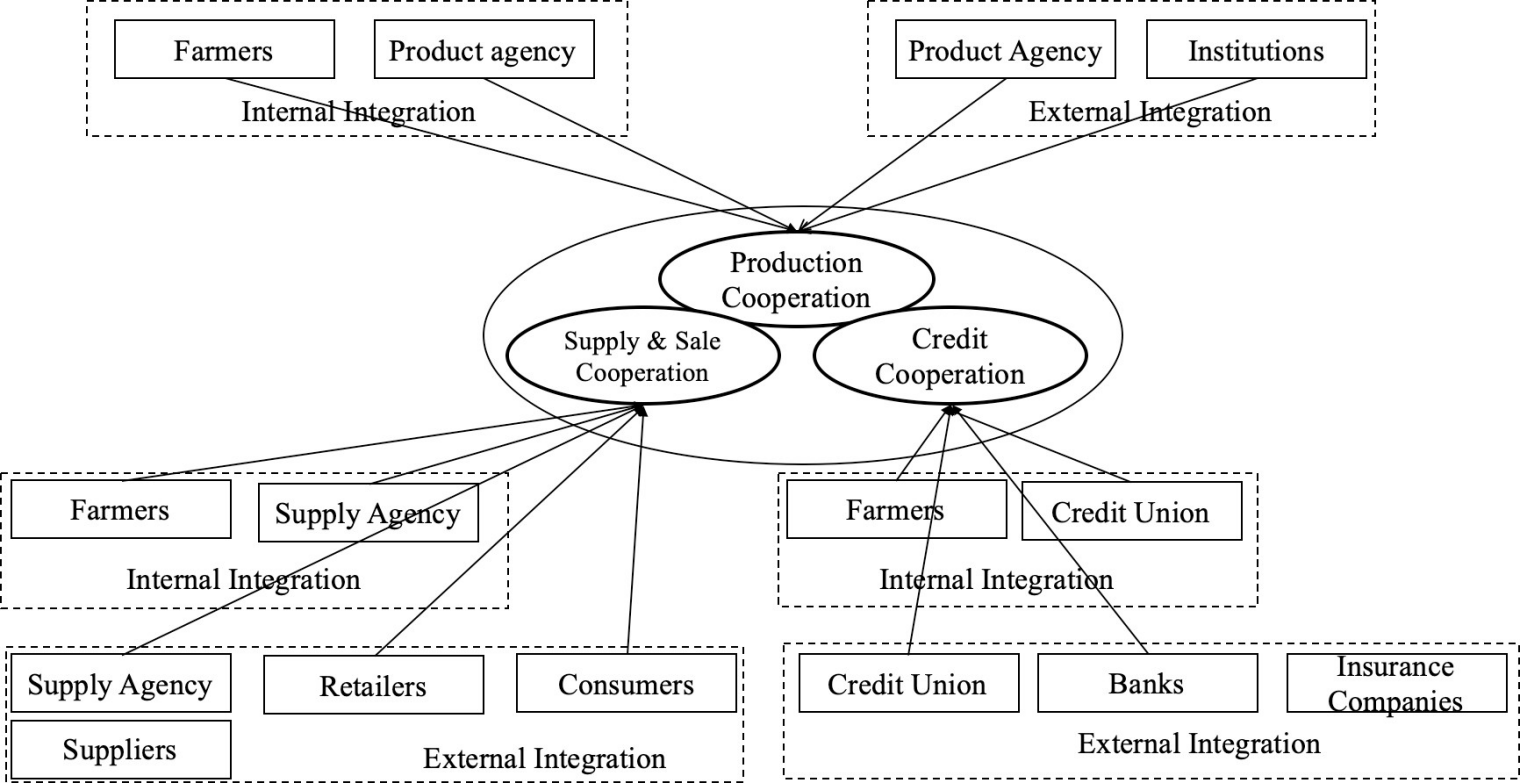
The Meiyu Cooperative’s trinity supply chain mode (based on internal and external supply chain integration theories).

Through internal integration and external integration in production cooperation, supply and sales cooperation, and credit cooperation, the Meiyu Cooperative integrates the trinity supply chain mechanism (see Fig 8), which effectively improves benefits for each actor in the actor network. From an environmental perspective, this trinity mechanism has successfully reduced pollution through a reduction in pesticides and ensured a green vegetable supply chain, which has improved vegetable safety across the agricultural industry.

## 5 Discussion

This study provides novel insights for supply chain integration and supply chain management in four dimensions as follows:

### 5.1 Internal integration

As mentioned above, internal integration means the extent to which firms transfer internal strategies, actions, and procedures to coordinate and thus meet other partners’ demands [30, 32, 34]. Previous articles explained that internal integration facilitates inner relationships through internal functional units in enterprises [40, 41], which is supported by this paper’s findings. Furthermore, our findings are consistent with research providing evidence for the foundational role played by internal integration in different sectors’ coordination in companies [41, 42]. The findings from this paper show that internal integration is more systematic for the Meiyu Cooperative than enterprises. For example, compared to enterprises, the Meiyu Cooperative establishes a stronger relationship not only with farmers in this cooperative but also with the Production Agency, Supply Agency, Wanke Company, and Credit Union to enhance coordination in its production, supply & sales, and financial processes. The stronger relationship achieves different interests based on actor network establishment. For instance, the Production Agency provides training opportunities for farmers to update their vegetable planting technology, thereby promoting production skills within the Meiyu Cooperative. Although one study suggested that internal integration does not completely improve shared information [43], the internal integration for the Meiyu Cooperative clearly explores and acquires a wide variety of information and resources.

In addition, we have found that the factors influencing internal integration on enterprises and professional cooperatives are not the same. Previous research demonstrates that enterprises’ internal integration is primarily influenced by partnership relationships and information sharing [44, 45]. By contrast, the Meiyu Cooperative in this paper is affected by its inner systematic organization through the Production Agency during the production process, the Supply Agency in the supply process, the Wanke Company during the sales process, and the Credit Union in the financial process. For example, farmers in the Meiyu Cooperative unified production standards through the Production Agency, which established a close network between farmers and the cooperative to improve productivity.

Because of this trinity of internal integration, compared to normal enterprises, the Meiyu Cooperative provides a stronger positive effect of internal integration and supports the necessary foundation for a higher level of integration with key shareholders. Thus, this professional cooperative operates more effectively than enterprises.

### 5.2 External integration

One study defined external integration as the degree to which enterprises establish intrastrategies, actions, and procedures to a coordinate level in order to conduct strategic alliances with other partners [24]. As expected, the findings in this paper demonstrate that external integration (with key suppliers) is critical in accelerating companies’ performance and capabilities [46, 47]. Similar to findings from one research [48], external integration for the Meiyu Cooperative involves interorganizational problem-solving based on actor network establishment. Specifically, it addresses suppliers’ needs for sustainable agricultural material orders. Additionally, it helps solve retailers’ problems in negotiating reasonable vegetable purchasing prices. Moreover, it helps meet customers’ demands for vegetable quality.

This study explains that the Meiyu Cooperative established a supply agency through supply cooperation with external suppliers. For example, the Supply Agency cooperated with the Wuzhou Fertilizer Group to collectively purchase more than 1,000 various agricultural materials at a comparatively low price through an external agreement. This, in turn, coordinates with the Wanke Company to improve sales coordination and integrate with external customers. For instance, the Wanke Company signed a contract in advance with the restaurant at Wenzhou Middle School to sell vegetables directly to students and teachers. In addition, the external integration of the Meiyu Cooperative expands its coordination in financial processes. For example, to increase external integration, the Meiyu Cooperative coordinates with the Rui’an Rural Commercial Bank based on a credit agreement that farmers can borrow money from the bank in advance to expand production and then return the money through the Meiyu Cooperative’s guarantee.

These findings support the research that external integration develops an effective mechanism to share information about product design, marketing plans, and delivery systems [49]. Through an organized network, clear interests from different sectors in the Meiyu Cooperative can reduce waste and inefficiency in the supply chain management process [50]. More importantly, further adding to the scholarship concerning external integration research, the external supply chain integration in this study expands specific dimensions regarding production, supply & sale, and credit process through the trinity mechanism.

### 5.3 Coordination between internal and external integration

The above findings indicate the demand for a cooperative to integrate both internal and external resources from different sectors inside and outside the cooperative, which means that internal integration and external integration in this paper mutually complement each other to establish a comprehensive network [33]. Furthermore, the trinity mechanism facilitates the harmony of internal and external integration during the production, supply & sale, and financial credit processes. Earlier literature—mostly qualitative and concerned with the management of supply chain integration—has explained the significance of communication and joint decision-making with different shareholders in various ways through the coordination of internal and external integration [34]. The findings in this paper are consistent with that argument. They also indicate that the Meiyu Cooperative, with a higher level of coordination through internal integration, would find it easier to cooperate with external partners to enhance external integration [34, 36, 44].

Additionally, this research finds that internal integration from the Product Agency in production cooperation, the Supply Agency during supply cooperation, the Wanke Company in sales cooperation, and the Credit Union during credit cooperation must all take place before external integration can be successfully implemented. For instance, in the sales process, the Meiyu Cooperative not only unifies vegetable prices for farmers through the Wanke Company to improve internal integration but also establishes a network with retailers such as supermarkets, wholesale markets, and middle schools to facilitate external integration. Thus, vegetables from the Meiyu Cooperative are competitive in the sales market. It would be interesting for future research to further explore this relationship between internal and external integration.

The findings in this paper indicate that professional cooperatives should not only pay attention to internal information sharing and collaboration among their sectors but also emphasize the significance of strategic collaborations with other shareholders outside the cooperative.

### 5.4 Economic, social, and environmental benefits

This new evidence is consistent with the statement concerning the different effects of internal and external integration [51]. Prior literature on supply chain integration has suggested that internal integration helps enterprises achieve operational benefits [7, 30, 34], while external integration facilitates the achievement of various objectives, such as product innovation [30, 52], quality management [53], and service delivery co-innovation [54]. Similarly, internal integration helps the Meiyu Cooperative to gain operational benefits, while external integration helps it achieve different sectors’ interests, such as product quality from customers and low cost for retailers, which clearly increases different partners’ economic benefits. This means that both enterprises and professional cooperatives have better financial performance as a direct result of supply chain integration (Table 1). More importantly, this paper provides additional interesting findings, as follows (Table 1). The trinity mode of the Meiyu Cooperative is positively associated with social performance [5] and environmental performance in rural China, which complements one research [37]. Specifically, in this paper, socially, the reputation of vegetables has increased due to skill improvement through internal integration and external integration in production, which satisfies customers. Moreover, environmentally, the trinity mode has facilitated sustainable development due to pollution reduction by internal and external integration in production skills improvement. Thus, strengthening internal and external integration should be given a high priority.

**Table 1:** The Meiyu Cooperative’s economic, social, and environmental performance since supply chain integration.

Dimension	The Meiyu Cooperative’s performance
Economics	• Revenue: increased from $460 (lower than the annual per GDP of Rui’an County) to $11,000 (higher than the annual per GDP of $10,000 in Rui’an County); • Farmers: grew from 94 up to 762; • Capital in Credit Agency: rose 231% from $769,200 to approximately $2,549,700.
Society	• Created a social impact on surrounding villages • Established well-known vegetable brands that increase consumer demand; • Attracted more talent; • Facilitated skills transfer from the Meiyu Cooperative to local farmers; • Tightened relationships between farmers and the Meiyu Cooperative.
Environment	• Lowered the use of pesticides/antibiotics; • Guaranteed green vegetables; • Facilitated safety and security of vegetables.

Utilizing actor network theory, this paper provides a comprehensive understanding of the trinity mechanism in enhancing supply chain integration. It expands supply chain integration theory by explaining how the trinity mechanism impacts different dimensions of internal and external integration. This study provides theoretical and empirical support for the formation of internal and external integration in different processes of professional cooperatives. Similar supply integration can aid different sectors in the supply chain to achieve various interests. This study enriches the research on supply chain management. The implications of professional cooperatives’ trinity supply chain mode with internal and external supply chain integration in production, supply & sale, and credit processes also contribute to future literature on supply chain integration.

## 6 Conclusion and implications

This paper reached several conclusions regarding the supply chain integration of the vegetable industry in China.

First, agricultural cooperatives in vegetable supply chains are important organizations that integrate different shareholders into the supply chains. Agricultural cooperatives collaborate and coordinate to innovate the trinity supply chain mode of supply chain integration in production cooperation, supply & sales cooperation, and credit cooperation. Second, coordination between internal and external integration in the production, supply & sale, and financial processes improves supply chain management in agricultural cooperatives. Third, all shareholders guided by the focal actor of the Meiyu Cooperative to form an actor network participating in internal and external supply chain integration benefit from the achievement of confirmed common interests.

The following suggestions result from our conclusions:

Agricultural cooperatives in the vegetable industry in rural China can contribute to supply chain integration, especially during this unique anti-COVID-19 period. The supply chain integration methods based on actor network theory examined in this research can serve as a model for organizations that do not have supply chain integration. To perform efficiently and effectively, organizations should do their best to collaborate and coordinate among different partners in their supply chain. To encourage collaboration and coordination among different shareholders through supply chain integration, the Chinese government might provide training opportunities and funding for supporting organizations to better understand how to coordinate within their supply chain so that the willingness of various shareholders to join the supply chain could also increase.

Our findings provide several important implications for future researchers. First, the results from this study show that coordination between different sectors contributes to supply chain integration. Future research may further explore the different levels of relationships between different sectors in supply chain integration, highlighting more specific operational outcomes at different levels of coordination. Second, the findings from this study establish a trinity supply chain mode based on actor network theory for agricultural cooperatives in China. Future research could elaborate on this administration model for other companies. For example, future research could examine the different factors affecting supply chain integration for companies in estimating the best practice. Third, this study only uses one case study, and it would be interesting to analyze more cases to analyze supply chain integration based on the actor networks established in agricultural cooperatives in China. Finally, this paper examines the trinity agricultural cooperative in the case of China, where this cooperative system has operated successfully. Thus, it is important to call attention to this successful operational experience of agricultural cooperatives, established by small-scale farmers in the rural economy in China, as a model for other countries.

## Supporting information

S1 File (PDF)

S1 Appendix (DOCX)

## References

[1] Rauta R, Gardasb B, Narwaneb V, and Narkhede B. Improvement in the food losses in fruits and vegetable supply chain—a perspective of cold third-party logistics approach. Operation Research Perspective. 2019; 1–13.

[2] Zhuo N, Ji C. Toward livestock supply chain sustainability: A case study on supply chain coordination and sustainable development in the pig sector in China. International Journal of Environment Research and Public Health. 2019; 16:3240–3256. doi: 10.3390/ijerph16183241 31487851 PMC6765892

[3] Wang L, Luo J. Vegetable supply chain integration: the case of a trinity cooperative in China. International Food and Agribusiness Management Review. 2019; 22 (5): 767–780. doi: 10.22434/IFAMR2019.0023

[4] Neszmélyi G. Family-based farming-can it be competitive? dilemmas on the farm-size in Hungary-successful patterns in the world: Denmark and South Korea. Taiwanese Agricultural Economic Review. 2017; 23 (2): 131–161

[5] Luo J, Guo H, Jia F. Technological innovation in agricultural cooperatives in China: Implications for agro-food innovation policies. Food Policy. 2017; 73: 19–33.

[6] Jia X, Huang J, and Xu Z. Marketing of farmer professional cooperatives in the wave of transformed agrofood market in China. China Economic Review. 2012; 23: 665–674.

[7] Wang Y, Dong W, Zhang Y, Su B, Chen Y. Analysis on agricultural products’ supply chain operation model in agricultural professional operatives. Rural Economy (Chinese Version). 2018; 4: 126–128.

[8] Ajates R. Agricultural cooperatives remaining competitive in a globalized food system: At what cost to members, the cooperative movement and food sustainability? Organization. 2020; 27 (2): 337–355. 10.1177/1350508419888900

[9] Zhao L. New Co-operative Development in China: An Institutional Approach. Institutes of International and European Policy. 2012. Catholic University of Leuven, Leuven, Belgium.

[10] Gao Y. Service function of farmer specialized cooperatives: theoretic and empirical study. Doctor Dissertation. 2014; 1–203.

[11] Huang Z, Gao Y. The realization degree and influence factors for Farmers’ Professional Cooperatives’ service functions. China Rural Economics (Chinese version). 2012: 4–17

[12] Tuna E, Kostas K. Agricultural cooperatives as social capital hubs–A case in a post-socialist country, Journal of Co-operative Organization and Management. 2021; 9 (1). 10.1016/j.jcom.

[13] Mikulcaka F., Haiderb L, Abson D., Newig J, Fischer J. Applying a capitals approach to understand rural development traps: A case study from postsocialist Romania. Land Use Policy. 2015; 43: 28–258.

[14] Vasa L, Baranyai Z, Kovacs Z, Szabo G. Drivers of trusts: Some experiences from Hungarian agricultural cooperatives. Journal of International Food & Agribusiness Marketing. 2014; 26: 286–297.

[15] Baranyai Z, Gyuricza C, Vasa L. moral hazard problem and cooperation willingness: Some experiences from Hungary. Aktualʹni Problemy Ekonomiky. 2012; 138: 301–310.

[16] Hansen MH, Morrow JL Jr, Batista JC. The impact of trust on cooperative membership retention, performance, and satisfaction: an exploratory study. The International Food and Agribusiness Management Review. 2002; 5(1), 41–59.

[17] Idris N, Arshad FM, Radam A, Ali NA. Agricultural cooperative supply chain management in Malaysia. Acta Hort. 2011; 895: 137–145.

[18] Ma W, Abdulai A, Goetz R. Agricultural cooperatives and investment in organic soil amendments and chemical fertilizer in China. American Journal of Agricultural Economics. 2018; 100 (2); 502–520.

[19] Zhang S, Sun Z Ma W, Valentinov V. The effect of cooperative membership on agricultural technology adoption in Sichuan, China. China Economic Review. 2020; 62:1–15.

[20] Forgács C. Leadership and Importance of Social Capital in Cooperatives during Transition: A Case Study of Two Cooperatives. Journal of Rural Cooperation. 2008; 36 (1): 57–72.

[21] Lee H. Oh S. A standards war waged by a development: Understanding international standard setting form the actor-network perspective. Journal of Strategic Information Systems. 2006; 15 (3):177–195.

[22] Brian S. Actor-network theory and stakeholder collaboration: The case of cultural districts. Tourism Management. 2005; 32 (3): 641–654.

[23] Uden L, Francis J. How to construct an actor-network: Management accounting from idea to practice. Critical Perspectives on Accounting. 2009; 21 (3): 243–251.

[24] Zhan A. Strategies on communication standardized based on actor network theory. Science Research(Chinese version). 2011; 29 (1): 56–62.

[25] Liu Z. “M-C-K” actor network model and the mechanism of knowledge production for interdisciplinary innovation team. Science and Science Technology Management (Chinese version). 2012; 33 (3):158–164.

[26] Wang C. The strategies for regional innovation based on actor network theory. The Progress of Science and Technology and Countermeasure (Chinese version). 2012; 29 (24): 52–55.

[27] Aka K. Actor-network theory to understand, track and succeed in a sustainable innovation development process. Journal of Cleaner Production. 2019; 225: 524–540.

[28] Callon M. 1986. Some elements of a sociology of translation: domestication of the scallops and the fishermen of St Brieuc Bay. P*ower, Action and Belief: A New Sociology of Knowledge* 196–223.

[29] Schoenherr T, Swink M. Revisiting the arcs of integration: cross-validations and extensions. Journal of Operation Management. 2012; 30 (1): 99–115. 10.1016/j.jom.2011.09.001

[30] Wiengarten F, Humphreys P, Gimenez C. Risk, risk management practices, and the success of supply chain integration. International Journal of Production Economics. 2016; 171–3, 361–370.

[31] Lee WC, Kwon LWG, Severance D. Relationship between supply chain performance and degree of linkage among supplier, internal integration, and customer. Supply Chain Management.2007; 12 (6): 444–452.

[32] Huo B, Cao Z, Li S, Zhao X. The fit between supply chain internal and external integration. System Engineering—Theory & Practice (Chinese version). 2016; 36 (2): 363–374.

[33] Wong C, Wong C, Boon-itt S. The combined effects of internal and external supply chain integration on product innovation. International Journal of Production Economics. 2013; 146: 566–574.

[34] Zhao X, Huo B, Selen W, Yeung J. The impact of internal integration and relationship commitment on external integration. Journal of Operations Management. 2011; 29 (1): 17–32.

[35] Hamprecht J, Corsten D, Noll M, Meier E. Controlling the sustainability of food supply chains. Supply Chain Management. 2005; 10: 7–10.

[36] Eksoz C, Mansouri SA, Bourlakis M, Önkal D. Judgmental adjustments through supply integration for strategic partnerships in food chains. Omega. 2019; 87, 20–33.

[37] Han J, Trienekens JH, Omta, S. Relationship and quality management in the Chinese pork supply chain. International Journal of Production Economics. 2011; 134: 312–321.

[38] Eisenhardt K. Building theories from case study research. Academy of Management Review. 1989; 14 (4):532–550.

[39] Yin R. Case Study Research: Design and Methods. 2003. Thousand Oaks: Sage Publications.

[40] Chen I, Paulraj A. Understanding supply chain management: Critical research and a theoretical framework. International Journal of Production Research. 2004; 42: 131–163.

[41] Jajja MSS, Chatha KA, Farooq S. Impact of supply chain risk on agility performance: mediating role of supply chain integration. International Journal of Production Economics. 2018; 205: 118–138. 10.1016/j.ijpe.2018.08.032.

[42] Cheng Y, Chaudhuri A, Farooq, S. Interplant coordination, supply chain integration, and operational performance of a plant in a manufacturing network: a mediation analysis. Supply Chain Management: International Journal. 2016; 21 (5), 550–568.

[43] Hillebrand B, Biemans WG. Links between internal and external cooperation in product development: an exploratory study. Journal of Product Innovation Management. 2004; 21: 110–122.

[44] Eksoz C, Mansouri A, Bourlakis M, Önkal D. Judgmental adjustments through supply integration for strategic partnerships in food chains. Omega. 2019; 87: 20–33.

[45] Wu L, Chuang CH, Hsu CH. Information sharing and collaborative behaviors in enabling supply chain performance: A social exchange perspective. International Journal of Production Economics. 2014; 148: 122–132.

[46] Tessarolo P, Is integration enough for fast product development? An empirical investigation of the contextual effects of product vision. Journal of Product Innovation Management. 2007; 24: 69–82.

[47] Sousa R, Da Silveira GJC. Capability antecedents and performance outcomes of servitization: differences between basic and advanced services. International Journal of Operation Production Management. 2017; 37 (4): 444–467. 10.1108/IJOPM-11-2015-0696

[48] Ragatz GL, Handfield RB, Petersen KJ. Benefits associated with supplier integration into new product development under conditions of technology uncertainty. Journal of Business Research. 2002; 55 (5) 308–400

[49] Lau KW, Tang E, Yam Richard CM. Effects of supplier and customer integration on product innovation and performance: Empirical evidence in Hong Kong manufacturers. Journal of Product Innovation Management. 2010; 27 (5): 761–777.

[50] Swink ML, Narasimhan R. Wang C. Managing beyond the factory walls: effects of four types of strategic integration on manufacturing plant performance. Journal of Operations Management. 2007; 25: 148–164.

[51] Wong C, Lai K, Cheng T. Value of information integration to supply chain management: role of internal and external contingencies. Journal of Management Information Systems. 2011; 28(3): 161–199.

[52] Jajja MSS, Kannan VR, Ali BS, Zahoor HS. Linkages between firm innovation strategy, suppliers, product innovation, and business performance: insights from resource dependence theory. International Journal of Operation Production Management. 2017; 37 (8): 1054–1075. 10.1108/IJOPM-09-2014-0424

[53] Zhao X, Wang P, Pal R. The effects of agro-food supply chain integration on financial performance: Evidence from Chinese agro-food processing business. International Journal of Production Economics. 2021; 231: 1–16.

[54] Tsou HT, Cheng CCJ, Hsu HY. Selecting business partner for service delivery coinnovation and competitive advantage. Management Decision. 2015; 53(9): 2107–2134. 10.1108/md-01-2015-0014

