# Supplementary material for: Agricultural cooperatives participating in vegetable supply chain integration: A case study of a trinity cooperative in China
Source: PLoS One. 2021 Jun 24;16(6):e0253668. doi: 10.1371/journal.pone.0253668 (PMC8224856; doi:10.1371/journal.pone.0253668)
Supplement: S1 File — (PDF) [file pone.0253668.s001.pdf]

interview transcripts

Information Statement

The Ethics Committee, School of Business, Wenzhou University supports the practice of protection for human subjects participating in research. The following information is provided for you to decide whether you wish to participate in the present study. You should be aware that even if you agree to participate, you are free to withdraw at any time without penalty.

We are conducting this study to better understand agricultural cooperatives in China. This will entail your completion of a interview. Your participation is expected to take about 30 minutes to complete. The content of the survey should cause no more discomfort than you would experience in your everyday life.

Although participation may not benefit you directly, we believe that the information obtained from this study will help us gain a better understanding of agricultural cooperatives in China. Your participation is solicited, although strictly voluntary. Your identifiable information will not be shared unless (a) it is required by law or university policy, or (b) you give permission.

Completion of the survey indicates your willingness to take part in this study and that you are at least 18 years old. If you would like additional information concerning this study before or after it is completed, please feel free to contact me by e-mail.

Sincerely,

Lu Wang

Principal Investigator

Assistant Profession, School of Business, Wenzhou University.

wanglu@wzu.edu.cn

Interviews

1st interviewee

Time: August 10, 2017

Location: the office for Meiyu Cooperative

Interviewer explained the information statement to interviewee.

Question: Could you please introduce the Meiyu Cooperative?

Answer: We are one of the top 100 National Agricultural Cooperatives in China. Meiyu Cooperative was established in April 2001. The membership has grew from 94 to 762 within 4,500 non-members. Also, members have expanded from 9 villages to 41 villages. Planting bases area has increased from 450 Chinese acres to 7,000 Chinese acres. The scope of business includes information technology, agricultural material procurement services, and agricultural product sales services providing for members. The annual sales income for Meiyu Cooperative has increased from 2 million CNY to more than 20 million CNY. And members' revenue has increased from 7,500 CNY to 18,000 CNY for each Chinese acre.

Question: Could you please tell us the management mechanism for Meiyu Cooperative?

Answer: This cooperative implements the management mechanism of the board of directors and the board of supervisors. There are 7 directors and 5 supervisors among them. Meanwhile, 4 talents coming from colleges and universities are introduced to the management level in order to seek common development. However, the internal board of directors and supervisors cannot actually make important decisions such as purchasing agricultural materials and agricultural product selling.

Question: How do members participate in the decision making process of Meiyu Cooperative:

Answer: The important decisions in the cooperative are jointly decided by the Member Congress. The Member Congresses are held two to three times annually. Each member has one vote in the decision making process. Decisions are made by the the majority of members' agreement. Members' opinions impact strongly on significant decision-making process.

Question: Could you please tell me honors that Meiyu Cooperative has won?

Answer: It was named National Farmer Cooperative Demonstration Cooperative in 2016.

Question： How does the Meiyu Cooperative cooperate with internal members.

Answer: Ideas provided by members should go through Member Congress. Members could communicate and share information with each other. Thus, members established a close relationship of mutual trust.

Question: Which external organizations does Meiyu Cooperative cooperate with?

Answer: It mainly cooperates with the local government, agricultural material suppliers, supermarkets, farmers' markets, schools, institutions, banks, insurance companies, etc.

Question: Could you please introduce that how Meiyu Cooperative established production cooperation?

In order to change the situation that traditional manual operation, extensive production management, and low working efficiency among small-scaled farmers, Meiyu Cooperative is committed to improve agricultural science and technology through implementing mechanical operation in cultivating, planting, and harvesting by operating with farmers. This operation could reduce manual input and increase output and efficiency. Furthermore, it improves large-scale operation. Through mechanized operation, the production cost reduced 500 CNY for each Chinese acre.

Question: Can you give me some details?

Answer: For example, Meiyu cooperative is committed to the application of new technologies in pesticide packaging recycling. This cooperative established a recycling station in Supply Agency to uniformly treat residual pesticides in order to prevent secondary pollution of the soil and water. In addition, this cooperative unified product testing standards to guarantee the quality and safety of agricultural products. It invested in the construction of pesticide residue testing station. Meanwhile, this cooperative allocated a full-time employee to conduct regular testing for agricultural products. Then, the test results would be announced to members timely to ensure that high-quality agricultural products entering the market. In addition, it cooperated with research institutes to build the Meiyu Agricultural Science and Technology Service Center in Ruian City. Meiyu Cooperative invited experts to local to provide professional services. Experts were invited to provide on-site service guidance 6 times and various on-site training courses were held three times last year. At the same time, online education programs were screened to provide different members. Finally, more than 300 members were trained and more than 400 copies of learning materials were handed out to farmers last year.

Question: Could you please tell me how Meiyu Cooperative establishes supply and sale cooperation?

Answer: We unified purchasing agricultural material to reduce production cost in supply cooperation. We have established the Supply Agency to responsible for the unified purchasing and supplying agricultural materials. The Supply Agency implemented group purchasing among members to directly group purchased from suppliers, which reduced the production cost and effectively eliminating the risk of members buying fake and shoddy products. Meiyu Cooperative provided a unified supply of 5 million CNY in agricultural supplies and rebated 100,000 CNY to members. This cooperative organized a group purchase involving 762 farmers of more than 100 tons of greenhouse film. The group purchase helped members save 300 CNY per ton comparing to market price.

For sale cooperation, Meiyu Cooperative integrate resource advantages and expand sales channels. In the beginning of Meiyu Cooperative, members were many, scattered, and most of them were aged traditional farmers. As a result, sales teams in this cooperative were not effective at the beginning. Therefore, Meiyu cooperative expanded sales channels such as enterprise, market, and super market. For instance, this cooperative has cooperated with large enterprises such as Ruian Huafeng Group and Tobacco Company. These large companies can place orders in advance and establish a vegetable distribution business, which greatly reduced the loss of intermediate links and brought fresh vegetables directly to the consumers' table. In this way, farmers would not plant blindly and the vegetable price would be more reasonable. It is natural for farmers to increase their revenue.

Question: Could you please tell me how the Meiyu Cooperative conducts credit cooperation?
Answer: Meiyu Cooperative together with other three agricultural cooperatives established Rui'an Humin Rural Capital Mutual Aid Agency approved by China Bank Regulatory Bureau to promote mutual finance among internal members. This mutual fund cooperative was registered by the capital of 5 million CNY and operated in a closed manner with 799 members. This mutual fund cooperative was based on agriculture, rural areas, and farmers to do mutual assistance and meet everyone's demand. Differ to other financial institutions, this cooperative formed a unique development service pattern. Till now, this cooperative has issued mutual fund of 325 million CNY to support hundreds of farmers in order to expand its business scale by 2, 800 Chinese acres. It achieved a second rebate for farmers of 350, 000 CNY.

Question: What you mentioned here was mutual financial assistance, were there any other credit cooperation examples?
Answer: Yes, insurance mutual support. Together with other 21 agricultural cooperatives and two natural persons, Meiyu cooperative jointed funding to establish Xingmin Rural Insurance Mutual Cooperative in Rui'an City, which became the first national pilot unit of property insurance rural insurance mutual cooperatives. It included 3, 552 members with a registered capital of one million CNY and operation capital of 5 million CNY. Three types of insurance such as agricultural product insurance, agricultural product freight insurance, and farmers' small loan guarantee insurance were developed around the agricultural production and operation. It has continually insured freeze injury insurance to cover 796.7 Chinese acres of greenhouse tomatoes with an insurance premium of 108 CNY per Chinese acre and the compensation as high as 1, 200 CNY.

Question: What do you think is your role in the Meiyu Cooperative?
Answer: As the previous Village Head, I mainly used my personal prestige to gain the members' trust and help them strengthen communication and increase mutual understanding. At the same time, I used my social resources to introduce external

cooperation to improve development of this cooperative.

2nd interviewee
Time: August 10, 2017
Location: the office for Meiyu Cooperative
Interviewer explained the information statement to interviewee.

Question: Do you think the role of farmers has changed during the development of Meiyu Cooperative?
Answer: Yes, I think so. I believe that as the major participant of this cooperative, farmers' role changed from the only labor input to both labor and investment together. In the past, farmers vegetable production, supply and sale mode was self-produced, self-sold. In this mode, farmer needed to complete multiple tasks such as production, processing, and sales independently. Through the support from trinity system from Meiyu Cooperative, farmers attended this cooperative and can choose to ransfer land to obtain subsidies and dividends. Meanwhile, farmers could select to engage in production under the guidance from this cooperative. The development of Meiyu Cooperative has broadened sale channels for farmers and helped them avoid the risk of delayed sales. Financing channels changed from single to diversity. In addition to dividends and planting income, farmers gained interest income through investing in the mutual fund cooperative and thus realized income diversification. Furthermore, farmers' loan changed from previous loaned from each other based on agricultural products to current loan from financial institutions based on their own credit through new financial system such as supporting from the mutual fund cooperative and rural commercial banks.

Question: Do you think the role of this cooperative has changed during the development process?
Answer: Meiyu Cooperative developed from limited participation to direct domination. This cooperative gradually changed from the role of limited participation to the role of domination in the process of production, supply and sale, and finance. At the same time, Meiyu Cooperative cooperated with other shareholders to establish common interests such as building a brand to enhance economic efficiency in sales. Specifically, this cooperative adopted a combination of online and offline sales to expand the sale market and drive the surrounding economy and thus achieve mutual benefit and mutual win cooperation. For mutual fund assistance, Meiyu Cooperative built a mutual financial assistance system. Specifically, Members voluntarily jointed and saved money in the mutual fund cooperative. Membersgot credit score and granted credit line to solve the problem of members' difficulty in loans to protect members' benefits. Besides, Meiyu Cooperative established its natural risk relief fund to provide compensation for members who would suffer in typhoon, and other disasters.

Question: What do you think are the major advantage of Meiyu Cooperative?
Answer: This cooperative could use various resources in production cooperation,

supply and sale cooperation, and credit cooperation. It brings more choices and opportunities through internal and external integration.

Question: Could you please tell me more details?
Answer: For example, we cooperated with Wenzhou Middle School. We can directly deliver vegetables from the fields to the restaurants in the school. On the one hand, we can ensure the food safety. On the other hand, the vegetable price for Wenzhou Middle school was relatively low through directly selling without any middle businessmen. Meanwhile, our cooperative can have stable customers to guarantee vegetable sale.

Question: What do you think is your contribution to the development of this cooperative?
Answer: I mainly inherited the good experience from the previous chairman and stabilized the development of the cooperative. Since I was a cashier, I paid more attention to the price. Thus I emphasized organizing members to link with farmers' markets. Our vegetable sales have been associated with farmers' markets in 17 cities across the country. Currently, our sale market are located in Hangzhou, Wuhan, Changzhou and other cities. The cooperative organized special sales to be responsible for distribution. More than 30 members worked as sales to expand more markets till now. We established Meiyu Agricultural Product Original Trading marked so that buyers did not need to compare prices and quality from door to door within opening bidding. The members who entered to this market can also achieve higher profits. At least, farmers can guarantee vegetable sales when vegetable prices fell.

3rd interview
Time: August 11, 2017
Location: the office for Meiyu Cooperative
Interviewer explained the information statement to interviewee.

Question: Could you please tell me what is the overall composition of Meiyu Cooperative?
Answer: This cooperative constituted with Production Agency, Supply Agency, Wanke Company which specialized in the marketing of agricultural products, Mutual Fund Cooperative, and Mutual Insurance Cooperative.

Question: What is the annual sale grow rate of your cooperative?
Answer: The income from sales has increased recent years and the increase is stable. And the total annual sale of our cooperative is greater than that of the same type of other vegetable agricultural cooperatives.

Question: I knew that your cooperative has your own brands. What role do these brands play in sales?
Answer: We have two major brands of *Qianglv* (Strongly Green) and *Lvyinxiang* (Green Impression). These brands has greatly enhanced the added value of vegetables from this cooperative. Thus, our cooperative has vigorously developed the local vegetable expanded business to other cities. Also, it cooperated with high-quality supermarkets to promote the business of high-quality vegetables. From this activity, our vegetable profits have increased by 4-5 times. Our cooperative achieved profit from distribution business of more than about 18 million CNY. Among these, vegetables from this cooperative sold to other cities was up to approximately 10 million CNY.

Question: Are all members' agricultural products handed to the cooperative for sales?
Answer: Some of members' agricultural products are sold through cooperatives, only 30 percentage.

Question: Do members need to pay membership dues to the cooperative?
Answer: Members do not need to pay membership dues.

Question: Is there any relationship between the annual revenue of the members and sales of the cooperative?
Answer: There is no direct relationship between members' annual revenue and sales of the cooperative.

Question: Has Wanke Company signed sale contracts with its members?
Answer: There is no sale agreements, but members' credit between Wanke Company and its members. Because sale contracts cannot limit members and it was not easy to

implement limitation for members.

Question: How is Meiyu Cooperative's bargaining power in the market?
Answer: This cooperative has some level of bargaining power in the market. For example, the Supply Agency realized the unified purchasing of agricultural materials, which could reduce the cost of agricultural materials to a certain extent. In addition, it increased the bargaining power in the purchasing of agricultural materials in the market.

Question: Compared to other agricultural companies, what are the advantages of Meiyu Cooperative?
Answer: Since this cooperative has an impact on the local area, they would have advantages in agricultural materials purchasing, agricultural products sales, and cooperation with government, colleges, and universities in agricultural technology training.

Question: What innovations does this cooperative have compared to other cooperatives?
Answer: There are three innovations: The first one is sale channel innovation through the establishment of Rui;an Original Agricultural Products Market and Wanke Company. The second innovation is the innovation of sales concepts through the registration of vegetable brands of *Qianglv* (Strongly Green) *and Lvyinxiang* (Green Impression) and insisting on the new sales concepts of *unity and mutual assistance, cooperation and mutual benefits.* The last one is to cooperate with research institutions to establish Rui'an Meiyu Vegetable Science and technology Service Center through inducing new seeds and new agricultural planting technologies

Question: What are the external organizations that Meiyu Cooperative cooperate with?
Answer: There are many types and numbers of organizations cooperating with this cooperative. And the cooperative conducted a fixed and mutual trust relationship with external organization through a long time operation. The main cooperation organizations include government, commercial banks, enterprises, research institutions, etc. As I mentioned above, we have many external organizations and the external network is large in scale. And our cooperative is the center of this social network and has a high degree of network openness.

Question: How do your cooperative gain external resources?
Answer: Overall, it was still difficult. The local government has lasted paying attention to the development of agricultural products and provided a large amount of financial funds in agricultural industry to promote farmers' revenue growth. Thus it is easy for our cooperative to apply funds from the local government. While there are still exiting difficulties in implement this beneficial policies. For example, if our cooperative want to get the agricultural subsidy, we would meet the standard from

this agricultural program. It is difficult to gain support from the local government through personal relationship of internal personnel of our cooperative. Thus, our ability to obtain resources from government is relatively weak. For banks, due to our reputation, it is easy to get support from local bank and our cooperation banks such as rural commercial bank. For enterprises and other external organizations, Meiyu cooperative signed sale contracts with other enterprises through Wanke Company since 2013. Also, we have stable agricultural suppliers. Besides, we cooperated with Wenzhou Science and Technology Vocational College to receive professional supports from experts.

Question: How do you think this cooperative get support from external resources and motivate internal members?
Answer: I believe it it mainly based on our common interests. Our original intention is to help everyone gain benefit. For example, we cooperated with research institution is to increase the added value of our vegetables and ultimately increase the market price. Members knew that we did everything for their sake, so they are cooperative. Research institution can also transfer their research into the reality. We cooperated with South Polar Science Investigation Team so that the team can get the high-quality vegetables and our cooperative have stable sale channels. Everyone gained benefits, thus we are willing to do cooperation.

Question: What do you contribute to the development of the cooperative?
Answer: As a college graduate, I came here and applied what professional knowledge I learned from the college into the Meiyu Cooperative, which brought new ideas and vivid. When I became the new chairman, the cooperative went several obvious changes in the past few years. First, the planting structure has changed from previous 80% of vegetables were greenhouse tomatoes to the current various local vegetables and became the largest perennial vegetable base in Wenzhou City. Second, our vegetables changed from previous large-scale sold to farmer market to curret deliver to canteens such as governments, hospitals, and large enterprises with high-quality. Third, our vegetables changed from previous no brand distribution to current *Lvyinxiang (Green Impression)* , which enhanced the value of local vegetables. Through quality monitoring and retrospective production, order-to-order production, and the *secondary rebates*, members' revenue has increased by more than 30%.

4th interview
Time: August 11, 2017
Location: the Supply Agency
Interviewer explained the information statement to interviewee.

Question: How do you attend this cooperative?
Answer: As Lei, Dafeng's younger sister, I came following my brother who felt this cooperative was pretty good to attend the cooperative.

Question: What do you do in this cooperative?
Answer: I worked as a clerk in the Supply Agency. I managed unified purchasing of agricultural materials. I could unified purchasing seeds, chemical fertilizer, etc. Then, members purchased agricultural materials from Supply Agency through me.

Question: What is your revenue?
Answer: 5, 000 to 6, 000 CNY per month.

Question: What is your source of revenue?
Answer: Mainly the wage income as a clerk for the Supply Agency.

Question: Have you attended to the Member Congress?
Answer: Yes, I have.

Question: Do you agree the decisions from the Member Congress?
Answer: Yes, I do.

Question: Do you think the Supply Agency provided supports for members?
Answer: Of course yes. I promptly shared the agricultural materials information with all members through Wechat group. It was definitely cheaper for members to uniformly purchase fertilizer from the Supply Agency compared with bought them separately. I would also let members to loan agricultural materials in advance if they were short of funds and they would return funds through selling out the vegetables later.

Question: What do you think of these three chairmen?
Answer: My elder brother is the current chairman of the cooperative. He improved our cooperative's reputation and increased our members' revenue since he because the chairman. I knew two previous chairmen after I arrived. The first chairman has a high prestige in our cooperative.

Question: Do you think attending the cooperative is a good thing?
Answer: Yes, I do. I followed my brother who helped the cooperative to be better than before to come here. I got a stable job. Everything is better and better.

Question: Do you think this cooperative would keep growing and developing and why?
Answer: The key is to get supports from the local government. If so, it would be definitely possible.

5th interview
Time: August 12, 2017
Location: the Supply Agency
Interviewer explained the information statement to interviewee.

Question: How did you attend this cooperative?
Answer: I was from Lishui City. Then I contracted some land and moved here. Chairman Huang introduced me to attend the cooperative. I believed his capability and trusted him. Thus it is good to attend the cooperative and produce under his leadership.

Question: What is your annual revenue?
Answer: Approximately 200, 000 annually.

Question: What is the source of your revenue?
Answer: Partly from the contracting of land, partly from the small business.

Question: Have you attended to the Member Congress?
Answer: Yes, I have.

Question: Did you agree the decisions from the Member Congress?
Answer: Yes, I did. Because the chairman discussed with us in advance and told us the intention for this decision making. We probably knew it was a good thing for all of us.

Question: Do you think the Supply Agency provided support to members?
Answer: I think it is good to be able to loan pesticides and fertilizers in advance with our credit. However, I would buy better and more expensive seeds else rather than in the Supply Agency. And for some new agricultural products, I would do the experiment first rather than adopt them directly on a large scale. By the way, the wechat group from the Supply Agency would provide some new information about agricultural materials.

Question: What do you think of these three chairman?
Answer:The previous chairman was able to distribute agricultural materials to members and communication between chairman and members. The capability for the 2nd chairman was weaker and he ignored to balance the distribution and relationship. The third chairman was introduced college graduate. He was motivated while the 1st chairman has a higher credibility in this cooperative.

Question: Do you think it is good to attend this cooperative?
Answer:It depends. It is cheaper to purchase seeds and fertilizers from the Supply Agency rather than purchase separately. It is useful to receive guidance from experts. However, mutual insurance cooperative is not efficient and was not able to enhance

members' ability to resist risks. Overall it is better to attend the cooperative.

Question: Do you think this cooperative would keep growing and developing and why?

Answer: As long as mutual agreement between members exsit and communication between each other is fluent, it is absolutely so. Of course, suggestions and demands from our members can be responded by the chairman and the cooperative at the beginning.

6th interview
Time: August 12, 2017
Location: the Supply Agency
Interviewer explained the information statement to interviewee.

Question: How did you attend this cooperative?
I'm one of the villager in this village. When the cooperative was established. I attended because everyone else attended.

Question: What is your annual revenue?
Answer: Approximately 80, 000 to 90, 000 CNY annually.

Question: What is the source of your revenue?
Answer: Mainly depending on the vegetable planting.

Question: Have you attended to the Member Congress?
Answer: Yes, I have.

Question: Do you agree with the decisions by the Member Congress?
Answer: Yes, I do agree. Because we would discuss them in advance.

Question: Do you think the Supply Agency provide support to members?
I think it is cheaper to buy fertilizers. And I can also review some information of seeds and agricultural tools from the wechat group. But if someone were not familiar with smart cellphone, it would be not easy for them to receive the information.

Question: What do you think of these three chairmen?
Answer: The first chairman was our previous Village Head who had a good relationship and communication with us. We also trusted him. The second chairman's capability was relatively ordinary. I did not know current one very well. It seems he did something new.

Question: Do you think it is good to attend this cooperative?
Answer: I think so. Everyone can express and communicate with each other. For example, it is convenient for one member to borrow money from the cooperative if he/she lacks money for greenhouse.

Question: Do you think this cooperative would keep growing and developing and why?
Answer: It is hard to say. It would always get better and better.
