# Supplementary material for: Agricultural cooperatives participating in vegetable supply chain integration: A case study of a trinity cooperative in China
Source: PLoS One. 2021 Jun 24;16(6):e0253668. doi: 10.1371/journal.pone.0253668 (PMC8224856; doi:10.1371/journal.pone.0253668)
Supplement: S1 Appendix — (DOCX) [file pone.0253668.s002.docx]

S1 Appendix

Table. List of six interviewees in the case study

| **Interviewees** | **Position** |
| --- | --- |
| Mr. Huang | First Chairman of the Meiyu Cooperative |
| Mr. Hong | Second Chairman of the Meiyu Cooperative |
| Mr. Lei | Third Chairman of the Meiyu Cooperative, manager of the Wenzhou Wanke Company |
| Ms. Lei | Clerk of the Supply Agency |
| Mr. Huang* | Member of the Meiyu Cooperative |
| Mr. Li* | Member of the Meiyu Cooperative |

Note. As requested, Mr. Huang and Mr. Li’s full names are disguised.
